# Development of Mechanistic In Vitro–In Vivo Extrapolation to Support Bioequivalence Assessment of Long-Acting Injectables

**DOI:** 10.3390/pharmaceutics16040552

**Published:** 2024-04-19

**Authors:** Daniela Amaral Silva, Maxime Le Merdy, Khondoker Dedarul Alam, Yan Wang, Quanying Bao, Nilesh Malavia, Diane Burgess, Viera Lukacova

**Affiliations:** 1 Simulations Plus, Incorporated, 42505 10th Street West, Lancaster, CA 93534, USA; maxime.lemerdy@simulations-plus.com (M.L.M.); viera.lukacova@simulations-plus.com (V.L.); 2 Division of Quantitative Methods and Modeling, Office of Research and Standards (ORS), Office of Generic Drugs (OGD), Center for Drug Evaluation and Research (CDER), U.S. Food and Drug Administration (FDA), Silver Spring, MD 20993, USA; khondoker.alam@fda.hhs.gov (K.D.A.); yan.wang3@fda.hhs.gov (Y.W.); 3 Department of Pharmaceutical Sciences, University of Connecticut, Storrs, CT 06269, USA; bqy.pharm@gmail.com (Q.B.); nilesh.malavia@uconn.edu (N.M.); d.burgess@uconn.edu (D.B.)

**Keywords:** long-acting injectable suspensions, PBBK, PBBM, complex generics, medroxyprogesterone acetate

## Abstract

Long-acting injectable (LAI) formulations provide sustained drug release over an extended period ranging from weeks to several months to improve efficacy, safety, and compliance. Nevertheless, many challenges arise in the development and regulatory assessment of LAI drug products due to a limited understanding of the tissue response to injected particles (e.g., inflammation) impacting in vivo performance. Mechanism-based in silico methods may support the understanding of LAI–physiology interactions. The objectives of this study were as follows: (1) to use a mechanistic modeling approach to delineate the in vivo performance of DepoSubQ Provera^®^ and formulation variants in preclinical species; (2) to predict human exposure based on the knowledge gained from the animal model. The PBPK model evaluated different elements involved in LAI administration and showed that (1) the effective in vivo particle size is potentially larger than the measured in vitro particle size, which could be due to particle aggregation at the injection site, and (2) local inflammation is a key process at the injection site that results in a transient increase in depot volume. This work highlights how a mechanistic modeling approach can identify critical physiological events and product attributes that may affect the in vivo performance of LAIs.

## 1. Introduction

Long-acting injectables (LAIs), often administered as a subcutaneous (SC) or intramuscular (IM) injection, are designed to provide sustained drug release over an extended period ranging from weeks to several months to improve product efficacy and safety, and patient compliance [1]. Considering this, LAIs are commonly used to treat a wide range of chronic disorders that require long-term therapy. This includes opioid use disorder, human immunodeficiency virus, cancer, schizophrenia, testosterone deficiency, and contraception [2]. 

Over the years, generic drug development of LAI drug products has remained a challenge. Many of these drug products have no/limited number of approved generic drug products, and this may directly impact the healthcare cost in the United States [3]. The lack of generics may be attributed to the complexity associated with product development and challenges for establishing bioequivalence (BE). Nowadays, in vivo BE study with pharmacokinetic (PK) endpoints is the most recommended approach to demonstrate BE between the reference standard and their generic drug product for LAI drug products. Some of the general challenges associated with conducting in vivo BE study include variability in PK parameters requiring a large number of subjects to establish BE, recruitment of patient population, and significant dropout rates, which may be due, at least in part, to the long time to reach steady state, the long washout period necessary, and the impact of early/delayed or missed doses resulting in long duration clinical studies [4]. To promote the generic development of LAI suspensions, efforts have been made to explore alternative BE approaches, such as the totality of evidence based on in vitro and/or in vitro/in vivo approaches, which require a mechanistic understanding of formulations’ characteristics and their in vivo performance. 

Physiologically-based pharmacokinetic (PBPK) modeling provides a unique opportunity to understand the in vivo mechanisms affecting an active pharmaceutical ingredient’s (API’s) local and systemic disposition and elimination; hence, it is a useful tool supporting the generic drug development process and approval [5,6,7,8]. Furthermore, physiologically-based biopharmaceutics modeling (PBBM) integrates in vitro dissolution and formulation characterization results into the model to predict a formulation’s in vivo behavior [5]. The PBBM approach can be used to establish in vitro–in vivo correlations (IVIVCs), allowing prediction of the PKs of formulation variants based on their in vitro dissolution profiles [6,7,8]. The establishment of mechanistic IVIVCs is a valuable approach that may enable the understanding of the impact of individual formulation attributes on the in vivo release behavior of LAI formulations. 

However, due to the complex formulation characteristics of LAIs, the physiological responses at the depot site, and the high variability in PK, establishing and validating IVIVCs remains a challenging task. To that end, recent research programs in this area (grant HHSF223201710135C; contract 75F40121C00133) have focused on promoting the development and application of mechanism-based modeling tools with the goal of facilitating the development of generic LAI formulations.

In the current study, Depo-subQ Provera^®^ 104, a medroxyprogesterone acetate (MPA) suspension, was selected as the model commercial product given the rise in LAI suspensions on the market [1]. The objectives of this study were to (1) use a PBPK modeling approach to mechanistically delineate the in vivo performance of Depo-subQ Provera 104 and formulation variants in preclinical species (rabbits), and (2) to predict human exposure based on the knowledge gained in rabbits.

## 2. Materials and Methods

### 2.1. In Vitro Characterization and Pre-Clinical In Vivo Studies

The formulation characterization of the reference listed drug (RLD) Depo-subQ Provera 104 and its formulation variants have been published previously [2,9,10]. Briefly, four LAI suspensions presenting a qualitatively and quantitatively (Q1/Q2) sameness to the RLD [11] were prepared that differed from the RLD in terms of API particle size or source of the formulation excipient PEG3350 (BASF and Spectrum chemicals). The pH of all formulations was 6.4, and they were composed of the following excipients: methylparaben, propylparaben, sodium chloride, polyethylene glycol, polysorbate 80, monobasic sodium phosphate⋅H_2_O, dibasic sodium phosphate⋅12H_2_O, methionine, povidone, and water for injection. In formulations F1 and F3, the API dispersed into the suspending media was used as received from the supplier; that is, the particle size was not changed as in formulations F2 and F4. Formulation variant F1 had a different supplier than F3. The API was recrystallized with an organic solvent in variant F2 and sonicated to obtain a smaller particle size in variant F4. Formulation variants and the RLD were characterized for particle size, morphology, and in vitro drug release performance [2,10]. The details of animal PK studies used to generate the MPA plasma concentration versus time profiles after subcutaneous and intravenous injections in rabbits have been described previously [10]. Briefly, all the formulations were injected subcutaneously into rabbits and blood samples were collected over a period of 110 days. Additionally, to characterize the systemic disposition of MPA in rabbits, a 4 mg MPA solution was injected intravenously [10].

### 2.2. Physiologically-Based Pharmacokinetic Model

A PBPK model for MPA was built using GastroPlus^®^ v. 9.8.3 (Simulations Plus, Inc., Lancaster, CA, USA). The model input parameters are listed in Table 1.

#### 2.2.1. Preclinical PBPK Model

A full-body PBPK model was used to describe the MPA systemic disposition in rabbits. Default values as generated by the GastroPlus algorithm for rabbit physiology were used for tissue sizes and blood flows. Due to lack of information about rabbit-specific tissue composition, the tissue composition parameters (fraction neutral lipid, fraction phospholipid, and fraction water content of plasma and tissue) were set to the same values as in the rat default GastroPlus PBPK physiology. A perfusion-limited model was used to describe the API distribution in all tissues. The tissue/plasma partition coefficients (Kp) were calculated using the default Lukacova method [17]. The kidney clearance was calculated as fraction unbound in plasma (Fup) * glomerular filtration rate (GFR) and the liver clearance was fitted so the total systemic clearance matched the in vivo clearance calculated from non-compartmental analysis (NCA) of the intravenous (IV) data [10]. The established PBPK model was then used to simulate MPA PK data obtained following SC administration in rabbits of Depo-SubQ Provera 104^®^ and formulation variants F1–F4 [10]. 

#### 2.2.2. Human PBPK Model

The human PBPK model was built using the same approach as the rabbit model: all tissues were described with a perfusion-limited model, the default Kp calculation method was used, renal clearance was defined as Fup*GFR, and the human liver clearance was informed by oral data [15]. To the best of our knowledge, there is no reported intravenous MPA administration in humans; hence, the baseline human PBPK model was validated with oral data obtained from the literature (Supplementary Material). The baseline PBPK model was then used to predict SC administration of the Depo-subQ Provera 104 formulation in healthy women. Injection site, dose, injection volume, and demographic information were selected according to the clinical or preclinical studies to inform model simulations [11].

### 2.3. SC Model

The Transdermal Compartmental Absorption and Transit (TCAT™) model, which also includes the SC compartment, was used to simulate MPA SC administration in both rabbits and humans. With SC administration, the drug is assumed to be injected into the extracellular space of the SC dosing compartment. The SC dosing compartment-depot is a small fraction of the adipose tissue, and its default volume is estimated as the volume of tissue where the extracellular space is large enough to accommodate the injection volume. 

According to the literature [18,19,20,21,22,23], following an SC injection there is a temporary increase in depot volume of approximately 3-fold because of the local inflammatory process [18,19,20,21,22,23]. The change in depot volume over time was incorporated into GastroPlus, as described by Equation (1).

(1) $$V_{depot,t}=V_{depot,0}\times \left(1+ScalingFactor\times Inflammation\right)$$

where V_depot,0_ and V_depot,t_ represent the volume of the depot compartment at time 0 and time t. Inflammation represents the magnitude of inflammation (i.e., the magnitude of depot volume increase) at time t, obtained from the Inflammation versus time profile (described in Equation (2)). The Scaling Factor is a parameter allowing for changes in the magnitude of inflammation along the entire inflammation versus time profile. The inflammation versus time profile was defined as a smooth function described by Equation (2):

(2) Inflammation=A×Exp(B×t)

where A and B are fitted parameters.

**Figure 1:**
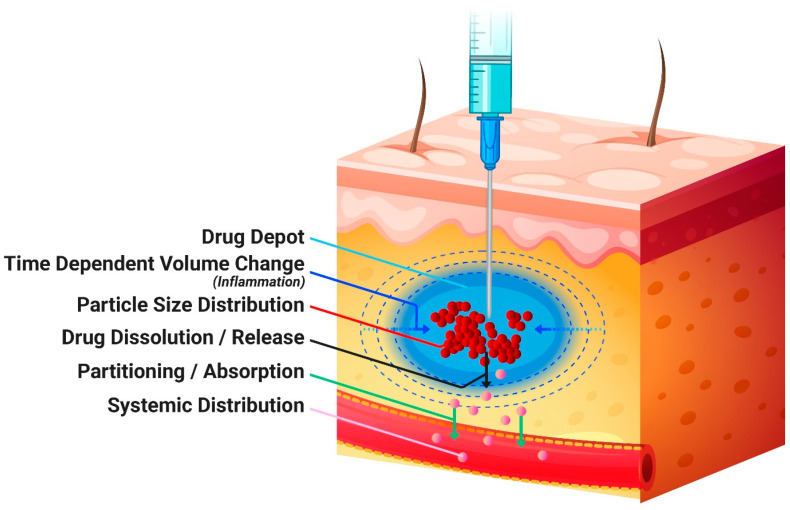
Conceptual diagram of a subcutaneous administration and systemic absorption.

After the administration of suspensions, the dissolution of the suspended API particles was modeled using the Johnson dissolution model [24]. Possible precipitation at the injection site was captured by the first-order precipitation model. The distribution of the dissolved API between the extracellular and the intracellular spaces of the dosing compartment was based on the model used for adipose tissue in the PBPK model and was described by an instant equilibrium since the perfusion-limited tissue model was utilized. From SC tissue, the dissolved drug entered the systemic circulation. This process is depicted in the diagram in Figure 1.

#### 2.3.1. Preclinical SC Model

A custom SC rabbit physiology model was defined with a tissue blood flow at the injection site of 16 mL/min/100 g tissue obtained from the literature [16]. The API percent bound to tissue in the SC dosing compartment was defined based on the API fraction unbound in the adipose tissue in the rabbit PBPK model (Table 1) [17].

The dynamic change in the depot volume is described by Equations (1) and (2). The inflammation parameters A and B in Equation (2) and the Scaling Factor in Equation (1) were fitted to the observed plasma concentration profile of the RLD product while keeping the maximum volume increase up to 3-fold, as reported in the literature [18,19,20,21,22,23]. The same depot volume versus time profile was subsequently used for all formulation variants.

The in vivo dissolution was simulated using the Johnson dissolution model [17]. The diffusion layer thickness and the in vitro–in vivo scaling of the MPA particle size distribution (PSD) were fitted to in vivo data, as described in Section 2.4. The same diffusion layer thickness and scaling factors for PSD were used across all formulations. 

#### 2.3.2. Human SC Model

Based on the clinical study protocol [11], the default abdominal human SC model in GastroPlus was used. The MPA-related parameters, such as fraction unbound in SC tissue and Kp, were defined based on parameters for adipose tissue in the human PBPK model (Table 1). Similar to the preclinical model, the dynamic change in the depot volume was included. The initial human SC model utilized the same inflammation versus time profile, inflammation scaling factor, diffusion layer thickness, and in vivo PSD as the rabbit SC PBPK model described above. Other scenarios were explored to consider interspecies differences [21,22] in the diffusion layer thickness (150 μm) and the magnitude of inflammation (Table 1). 

### 2.4. In Vitro and In Vivo PSD

The API PSDs in model inputs were described by a log-normal distribution, where the mean radius and standard deviation (SD) were fitted to the experimentally obtained in vitro PSD data for each MPA formulation [10]. The cumulative PSD was fitted using a built-in tool in GastroPlus against Dv10, Dv50, and Dv90 values, which represent the size below which 10%, 50%, or 90% of all particles are found, respectively. The in vivo PSD, used to parameterize the in vivo dissolution component of the model after SC injection, were calculated by increasing the in vitro mean radius and SD by 1.8- and 4.8-fold, respectively, for each formulation (scaling factor). The minimum radius in the in vivo PSD was set to half of the in vitro mean radius. The scaling factors for mean radius and SD as well as cutoff for the minimum radius in the PSD were obtained by fitting to the plasma concentration vs. time profile for the RLD product. Since the variants were Q1/Q2 formulations, it was assumed that the aggregation process would be similar between all formulations. Hence, the same scaling factors were applied to determine the in vivo PSD for all formulations. As shown in Table 2, the final PSD depends on the initial (measured) PSD, which means that while the same scaling factors were used each formulation will have a different in vivo PSD. The in vitro and in vivo PSDs for all formulations are summarized in Table 2. 

Additionally, due to the more static environment in the SC compartment, it was assumed that the diffusion layer thickness is higher [25] than the default value in GastroPlus^®^ (maximum value of 30 μm), which is typically used for orally administered compounds. Therefore, the diffusion layer thickness was increased in the SC injection simulations (80 μm and 150 μm for rabbit and human, respectively).

### 2.5. Acceptance Criteria for Model Validation

The predictions were considered accurate when the shape of the simulated plasma concentration vs. time (C_p_-time) profile closely matched with the shape of the average observed C_p_-time profile and the prediction errors for C_max_ and AUC_0-t_ were within ±25%. The predictions were considered acceptable when the predicted C_max_ and AUC_0-t_ were within 2-fold of the observed values. 

## 3. Results

### 3.1. Preclinical PBPK Model

**Figure 2:**
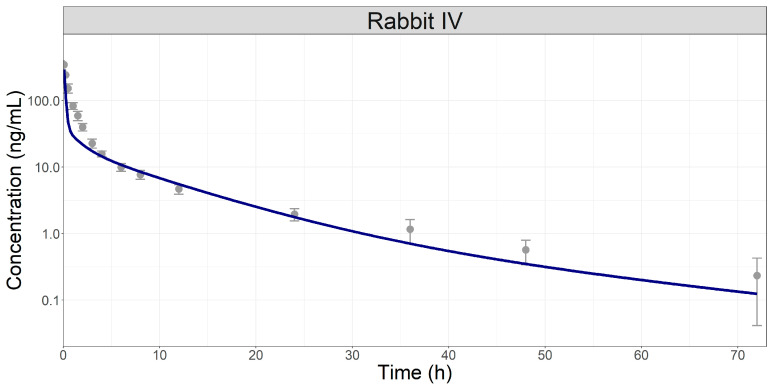
Simulated (line) and observed (symbols) plasma concentration-time course following a single 4 mg IV bolus administration of MPA in rabbits [10].

The volume of distribution (Vd) of 51.17 L predicted by the PBPK model was comparable to the Vss from NCA of the average Cp-time profile after IV administration in rabbits (67.95 L). The liver clearance (8.823 L/h) was defined based on the NCA analysis after adjusting for renal elimination defined as Fup*GFR. Using these model settings, the plasma concentration-time course of MPA following IV administration in rabbits was adequately described by the model, as shown in Figure 2. 

### 3.2. Preclinical SC Model and In Vitro–In Vivo Extrapolation

**Figure 3:**
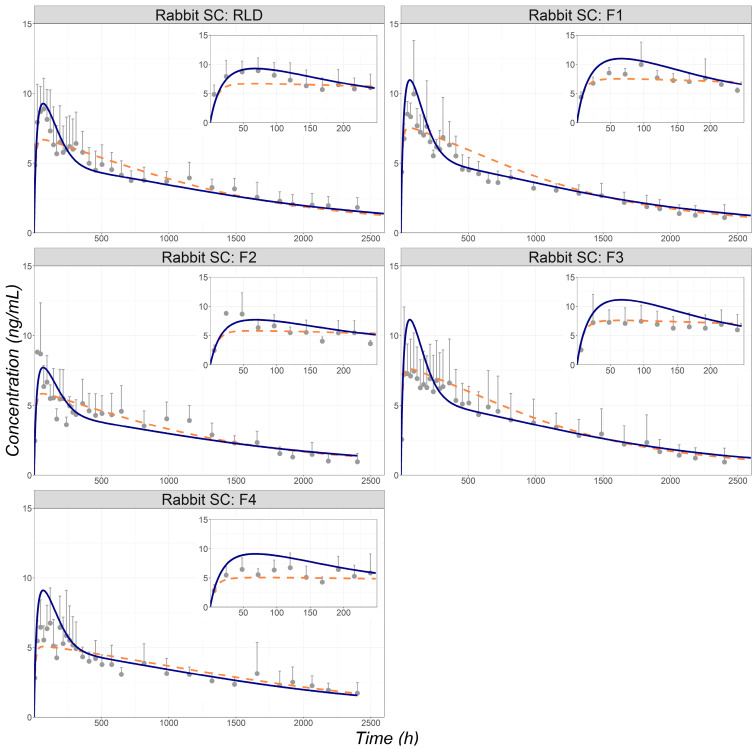
Simulated (lines) and observed (symbols) plasma concentration time course following 104 mg SC administration of MPA as the LAI RLD and four formulation variants (F1 to F4) in rabbits [10]. Predictions are based on PSD using the same scaling factors across all formulations. Dashed orange line: plasma profile with no inflammation considered; Solid blue line: plasma profile with inflammation included in the model. RLD: reference listed drug.

Simulation without inflammation, using the experimental PSD and the default diffusion layer thickness to describe the MPA in vivo dissolution after SC injection, resulted in misprediction of the MPA C_p_-time profile for all formulations (Supplementary Material—Figure S1). Therefore, a methodology was developed to account for in vivo dissolution of MPA from different formulations and consisted of changes to diffusion layer thickness, PSD, and effective depot volume. The increase in diffusion layer thickness (80 μm and 150 μm for rabbit and human, respectively) was not unexpected considering that the upper limit of 30 μm (default value) is based on in vitro dissolution experiments with well-stirred media [24]. However, changing diffusion layer thickness alone was not sufficient to capture the shape of the Cp-time profiles and a change in PSD was necessary. The scaling factors for mean and SD in PSD were fitted to improve the description of the Cp-time profile shape (especially at the later times) for RLD, and the same scaling factors were used to estimate in vivo PSD from measured in vitro distributions for all formulations. The fitted diffusion layer thickness and the PSD scaling were critical to capture the extended terminal phase in the Cp-time profiles. To accurately capture the C_max_ and the shape of the Cp-time profile within the first two weeks after injection, a time-dependent change in the depot volume due to inflammation was incorporated into the model. The simulated MPA Cp-time profiles simulated by models with and without time-dependent change in the depot volume are compared in Figure 3. The observed and simulated C_max_ and AUC_0-t_ values are summarized in Table 3. The simulated AUC_0-t_ values were within ±25% of the observed values for all formulations with or without including the time-dependent change in the depot volume due to inflammation. The C_max_ values were mostly underpredicted when inflammation was not included and predicted accurately (within ±25%) or overpredicted (fold-error more than 25%) when inflammation was included. Nevertheless, the predicted PK endpoints ranged from 0.88- to 1.5-fold of the observed values when inflammation was included. The inclusion of inflammation parameters (resulting in the dynamic change in the depot volume) also significantly improved the description of the observed profile shapes. It is worth noting that formulation F3 did not present the initial higher absorption rate, and the increase in the initial absorption rate for formulation F4 appeared to be lower than observed in the other formulations. Different sources of excipient in formulation F3 and stability issues (change in particle size upon storage) in formulation F4 may be responsible for the differences observed for these two formulations. However, direct data are not available to confirm or further evaluate this hypothesis. 

### 3.3. SC Model in Humans

The ability of the human PBPK model to predict MPA systemic distribution and elimination was validated using oral data obtained from the literature [15]. The model accurately described MPA clinical PK following oral administration of multiple drug products with predicted C_max_ and AUC_0-t_ within 0.79- to 1.2-fold of the observed values across three different studies (Table S1). Additional details are presented in the Supplementary Material. This model was subsequently used to describe MPA clinical PK following SC administration.

**Figure 4:**
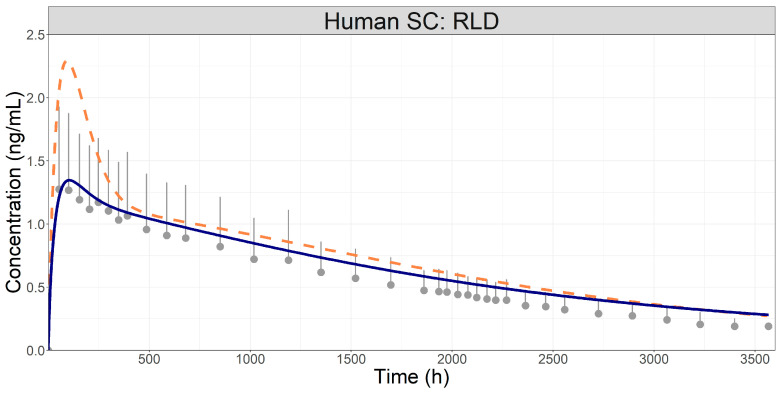
Simulated (lines) and observed (symbols) plasma concentration time course following SC administration of Depo-subQ Provera 104^®^ in humans [11]. Dashed orange line: predictions with the same parameters as used in the rabbit model; Solid blue line: Adjusted diffusion layer thickness and inflammation scaling factor.

The initial prediction of the human PK after SC administration of the RLD was performed using the same diffusion layer thickness, inflammation profile, and in vivo PSD for the RLD, as determined in the preclinical SC model. As the RLD product was used in both species, it was anticipated that the formulation properties were the same. As shown in Figure 4, this approach resulted in an overall overprediction of plasma concentrations, especially around the C_max_, even though the predicted C_max_ and AUC_0-t_ were within 2-fold [26] of the observed data (Table 4). The dissolution process in humans seemed to be slower than in rabbits, which was captured by further increasing the diffusion layer thickness and decreasing the overall degree of inflammation (Figure 4). These parameters are linked to the physiological response to the injection at the depot site (therefore may be different between rabbits and humans), their adjustment resulted in a closer match to the profile shape, and both the C_max_ and AUC_0-t_ predictions were within ±25%.

## 4. Discussion

The SC injection site is located below the dermis and is composed of loose connective tissue and adipose tissue that is permeated by blood capillaries (Figure 1) and a lymphatic capillary bed [27]. Such characteristics make the SC space an ideal route of administration for sustained systemic drug exposure of sparingly soluble compounds. The release mechanism of LAI suspensions is controlled by the physicochemical properties of the API (such as solubility in the surrounding fluid and accessible surface area) as well as the formulation composition and physicochemical characteristics [1]. An increasing surface area to volume ratio obtained with decreasing particle size is expected to result in faster API dissolution. This was observed in the in vitro dissolution studies for the Q1/Q2 formulations reported by Bao et al., 2022 [9]. However, the particle size to dissolution rate relationship was not observed in vivo to the same extent as in the in vitro experiment. The interactions between LAI formulations and the physiological environment at the depot site are not fully understood and additional methods are needed to mechanistically predict the local drug dissolution and absorption into the systemic circulation based on the drug products’ physicochemical characteristics. As part of those efforts, the current study has evaluated the use of PBPK modeling informed by in vitro formulation characterization to mechanistically understand the in vivo performance of SC-administered MPA suspensions in rabbits and extrapolate these findings to humans.

The preclinical PBPK model calibrated against rabbit IV data was used to predict the plasma concentration profile of MPA following SC administration in rabbits based on the in vitro formulation characterization data for the RLD and four formulation variants. Since the distribution and elimination were well captured with the IV data, any discrepancy between observed and predicted SC profiles would be attributable to the dissolution of MPA at the injection site; therefore, parameters such as PSD and depot volume were fitted. 

The first step in assessing the bio-relevance of the in vitro measurements is to use them as a direct input in the PBPK model. The in vitro-measured particle size and MPA solubility led to a misprediction of C_p_-time profiles for all formulations (Supplementary Material—Figure S1), suggesting additional mechanisms need to be considered in the model to reflect the processes that the formulations are undergoing in vivo at the depot site. One of the events that particles may undergo in the SC space is aggregation due to the restricted tissue environment [25,28]. Particle aggregation causes the effective in vivo particle size (particle size representing the dissolution surface area of the aggregate) to be greater than the in vitro-measured data. This hypothesis was tested in the model by scaling the in vitro PSD (Table 2). The injection method, applied shear specifically, may also impact the effective particle size driving the dissolution rate, as reported by Smith et al. [25]. Interestingly, the scaling factor for the mean particle radius fitted in the present study is comparable to the average 1.82-fold increase in D_50_ values with low shear compared to high shear in the Smith study [25]. However, to correctly match the shape of the observed MPA C_p_-time profiles from all formulation variants, not only mean but also standard deviation of the PSD had to be increased from the in vitro-measured values, suggesting the influence of other factors on the dissolution rate than injection method alone. The final in vivo distribution included a portion of extremely large particles, which may suggest continued aggregation over time at the depot site due to the limited volume. There is no direct measurement to support this hypothesis at this stage; however, the approach utilized to estimate the in vivo PSD s was able to describe reasonably well the C_p_-time profiles across all tested formulations. As the RLD and its formulation variants administered in the preclinical study were Q1/Q2 the same, the aggregation processes were assumed similar between all formulation variants. 

In addition to drug product-related processes that may occur during and after the injection, physiological reactions at the depot site must be considered. The deposition of foreign materials in the SC space can result in a variety of tissue responses, often characterized as an injection site reaction [29]. As described in the literature, the inflammatory response to an injected material can lead to a transitory increase in the depot volume [18,19,20,21,22,23]. Jucker et al. characterized the depot kinetics of cabotegravir long-acting formulation in both rats and humans [21,22]. In both species, there was an increase and subsequent decrease of the depot volume, which was well correlated to the plasma drug concentration. As the formulation, compound, and the preclinical species studied by Jucker et. al. were different from this study, the inflammation profile was not used directly, but the extent of the inflammation (up to a 3-fold increase in the depot volume) was assumed to be similar. The transient depot volume increase can lead to a higher initial dissolution rate, which explains the observed faster rise in the plasma concentration in the first days after injection. As the inflammation subsides, the volume decreases, and a more sustained drug release is obtained. The inclusion of inflammation in the model resulted in an improved prediction of the plasma concentration profile shapes. As reported by Paquette et al. [29], there were key differences in the foreign body reaction at the injection site after SC injection of two poorly soluble compounds suspended in the same injection vehicle when their PSDs were matched, indicating that the tissue responds in a distinct manner to each compound. Hence, the same inflammation profile was assumed for all formulation variants as the same compound was administered while the impact of particle size was considered partially within the context of particle aggregation, as discussed above. 

The impact of particle size on the inflammatory reaction at the depot site has been previously reported [30]. However, with expected significant inter-individual variability due to fluctuation in the immune response for the different rabbits in the study, a formulation-specific inflammation based on particle size was not integrated into the model at this stage. We acknowledge that the model has limitations given that different particle sizes can show different behavior to the same shearing stress and lead to different levels of inflammation post administration, which may subsequently lead to a difference in aggregation and dissolution rate. More studies with different APIs and formulation variants (PSDs, API solubility) that show higher sensitivity to these mechanisms are needed to investigate their relative contribution to the drug release and the extent of inflammation observed in vivo before these processes can be mechanistically captured into future iterations of the PBPK model developed here. 

Once the preclinical PBPK and SC absorption model was established and validated, it was translated to a human SC PBPK model. Human physiologies were generated using the built-in GastroPlus algorithm. The human PBPK model was verified with oral data (Supplementary Material) confirming that it adequately described the systemic disposition of MPA in humans before it was used to simulate SC administration. The assumption made in the preclinical model stands for human prediction: once the distribution was well characterized, discrepancies between observed and predicted SC profiles would be attributable to the dissolution/absorption from the depot site. Using the same formulation and inflammation parameters as in the preclinical model generally overpredicted the human pharmacokinetics after SC administration (Figure 4), but the predicted C_max_ and AUC were still within the generally accepted 2-fold range [26]. These results highlight the usefulness of PBPK modelling for interspecies scaling to make an early prediction of the pharmacokinetics in humans. 

Inflammation is an important process that may affect the dissolution kinetics at the depot site and, consequently, the absorption into the systemic circulation [31,32]. Due to physiological differences, the inflammatory process may differ between rabbits and humans. For example, the higher SC blood flow rate in rabbits compared to humans could result in higher inflammatory cell recruitment and oedema, leading to a more exacerbated inflammatory response. Hence, the inflammation profile in the human SC model was decreased in magnitude while keeping the same profile shape. Furthermore, the diffusion layer thickness was changed to a higher value. The possible reasons for this still need to be investigated. Modifying both the inflammation factor parameter and the diffusion layer thickness improved the predictions with both C_max_ and AUC_0-t_ within BE limits.

We acknowledge that the SC PBPK model built based on complex animal data was only verified for RLD (Depo-subQ Provera 104) in humans. The available data in the literature were leveraged to conduct this study, and no fit-for-purpose study was conducted. Hence, the assessment of formulations F1–F4 using the built SC PBPK model was performed only for rabbits.

## 5. Conclusions

The SC PBPK models developed here captured well the systemic MPA exposure in rabbits and humans following the administration of the RLD and carefully selected formulation variants by accounting for formulation attributes such as PSD, MPA solubility, and formulation-physiology interplay at the injection site (i.e., inflammation). The validated model was able to describe the impact of physiological differences between species on the inflammation process and API dissolution at the injection site. The work presented herein highlights how mechanistic modelling approaches allow the identification of physiological events and product attributes that will be critical for the in vivo performance of LAIs. Hence, the assessment of in vitro–in vivo extrapolations is the first step in paving the way to create mechanistic IVIVCs. Further studies investigating the physiological events at the depot site, interspecies differences, and in the vivo-relevant drug product performance of additional drugs are needed to improve model predictions and establish the role of modeling in the development of LAI drug products. 

## Figures and Tables

**Table 1:** Summary of PBPK input parameters of MPA.

Input Parameter	Value	Unit	Source
***MPA physicochemical properties***			
Molecular weight	386.53	g/mol	ADMET Predictor ^a^
Solubility @ pH 7.4	0.27	μg/mL	[2]
Suspension solubility	0.01	mg/mL	[2]
pKa	N/A (Neutral compound)		
LogP	3.82		[12]
Mean precipitation time	900	s	GastroPlus^®^ default
Diffusion coefficient	0.62	cm^2^/s × 10^−5^	ADMET Predictor
Human jejunal permeability	7.307 × 10^−4^	cm/s	ADMET Predictor
Drug particle density	1.2	g/mL	GastroPlus^®^ default
Blood/plasma concentration ratio	0.83		ADMET Predictor
Fraction unbound in plasma	12	%	[13,14]
Kp prediction method	Lukacova		Calculated default GastroPlus^®^ method ^b^
***Clearance***			
Clearance mechanism	Metabolic (ECCS)		[13]
Kidney CL (Rabbit/Human)	0.056/0.842	L/h	Calculated as Fup*GFR
Systemic liver CL (Rabbit/Human)	8.78/30.6	L/h	Fitted to rabbit intravenous PK data [10]/informed by oral data [15]
***Subcutaneous parameters***			
Injection volume ^c^	0.64	mL	
Effective depot volume (Rabbit/Human)	3/4.74	mL	Fitted
Fraction unbound in tissue (Rabbit/Human)	0.1/0.2	%	Calculated default GastroPlus^®^ method ^c^
Blood flow rate (Rabbit/Human)	16/3.77	mL/min/100 g SubQ	[16]/GastroPlus^®^ default
Inflammation scaling factor (Rabbit/Human)	0.536/0.03		Fitted
Inflammation lag	0	day	Fitted
Inflammation A	5.583		Fitted
Inflammation B	0.409		Fitted
Diffusion layer thickness (Rabbit/Human)	80/150	μm	Fitted

^a^ Predicted from structure by ADMET Predictor^®^ v10.4 (Simulations Plus, Inc.); ^b^ Both K_p_ and F_ut_ calculations are based on drug (logP, pKa, adjusted fraction unbound in plasma (Fup), and blood/plasma concentration ratio (R_bp_)) and physiological (tissue composition) properties; ^c^ the same injection volume for RLD and formulation variants. CLint: intrinsic clearance; Fup*GFR: plasmatic fraction unbound * glomerular filtration rate; ECCS: extended clearance classificationsSystem: SubQ: subcutaneous.

**Table 2:** In vitro–in vivo particle size scaling.

Formulation	In Vitro PSD [10]		In Vivo PSD		
	Mean ^#^ Radius (μm)	SD ^#^ (μm)	Mean ^#^ Radius (μm)	SD ^#^ (μm)	Rmin (μm)
RLD	9.06	3.81	16.30	18.34	4.53
F1	6.67	3.14	12.00	15.06	3.33
F2	10.47	6.20	18.84	29.78	5.23
F3	6.48	3.06	11.67	14.67	3.24
F4 *	9.39	3.86	16.92	18.51	4.69

^#^ Mean and SD represent parameters in log-normal distribution function. * Formulation F4 was not stable upon storage. The particle size was monitored over time and the plateau value was used in the model.

**Table 3:** Comparison of C_max_ and AUC_0-t_ fold-errors after SC administration of the four test formulations and the RLD.

	RLD			F1			F2			F3			F4		
	Obs.	Sim.	FE	Obs.	Sim.	FE	Obs.	Sim.	FE	Obs.	Sim.	FE	Obs.	Sim.	FE
**Scaled diffusion layer thickness and PSD**															
C_max_	8.9	6.69	0.75	9.96	7.53	0.76	8.82	5.84	0.66	7.46	7.61	1.02	6.75	5.07	0.75
AUC_0-t_	9229	9053	0.98	8435	9478	1.12	7659	7556	0.99	8714	9527	1.09	7779	8088	1.04
**Scaled diffusion layer thickness and PSD & Inflammation**															
C_max_	8.9	9.28	1.04	9.96	10.98	1.1	8.82	7.72	0.88	7.46	11.13	1.49	6.75	9.107	1.35
AUC_0-t_	9229	8730	0.95	8435	9193	1.09	7659	7273	0.95	8714	9245	1.06	7779	8343	1.07

C_max_ in ng/mL; AUC in ng·h/mL; RLD: reference listed drug; FE = fold-error (simulated/observed).

**Table 4:** PBPK model prediction statistics for the RLD following SC administration in humans.

		Observed [11]	Simulated	FE
Same as rabbit	C_max_	1.276	2.289	1.79
	AUC_0-t_	2005.5	2680	1.34
Updated model	C_max_	1.276	1.347	1.06
	AUC_0-t_	2005.5	2360.8	1.18

C_max_ in ng/mL; AUC in ng-h/mL; FE = fold-error (simulated/observed).

## Data Availability

The data presented in this study are available in this article and Supplementary Material.

## Supplementary Materials

The following supporting information can be downloaded at: https://www.mdpi.com/article/10.3390/pharmaceutics16040552/s1, Figure S1: Plasma concentration time profile following SC administration of MPA LAI in rabbits. Predictions based on measured particle size and no inflammation; Solid line: predicted. RLD: Reference Listed Drug; Figure S2: Observed (squares) and simulated (line) plasma MPA concentration after administration of 500 mg Farlutal^®^ tablet (A), 500 mg Provera^®^ tablet (B), and 500 mg Granule formulation (C) in men; Table S1: Comparison of observed and predicted Cmax and AUC after oral MPA administration.
