# Supplementary material for: Development of Mechanistic In Vitro–In Vivo Extrapolation to Support Bioequivalence Assessment of Long-Acting Injectables
Source: Pharmaceutics. 2024 Apr 19;16(4):552. doi: 10.3390/pharmaceutics16040552 (PMC11054330; doi:10.3390/pharmaceutics16040552)
Supplement: Supplementary file 1 [file pharmaceutics-16-00552-s001.zip › Supplementary material-Figure S1.pdf]

Preclinical predictions - Subcutaneous administration with experimental particle size

Figure S1. Plasma concentration time profile following SC administration of MPA LAI in rabbits. Predictions based on measured particle size and no inflammation. Empty squares: observed data (1); Solid line: predicted. RLD: Reference Listed Drug.

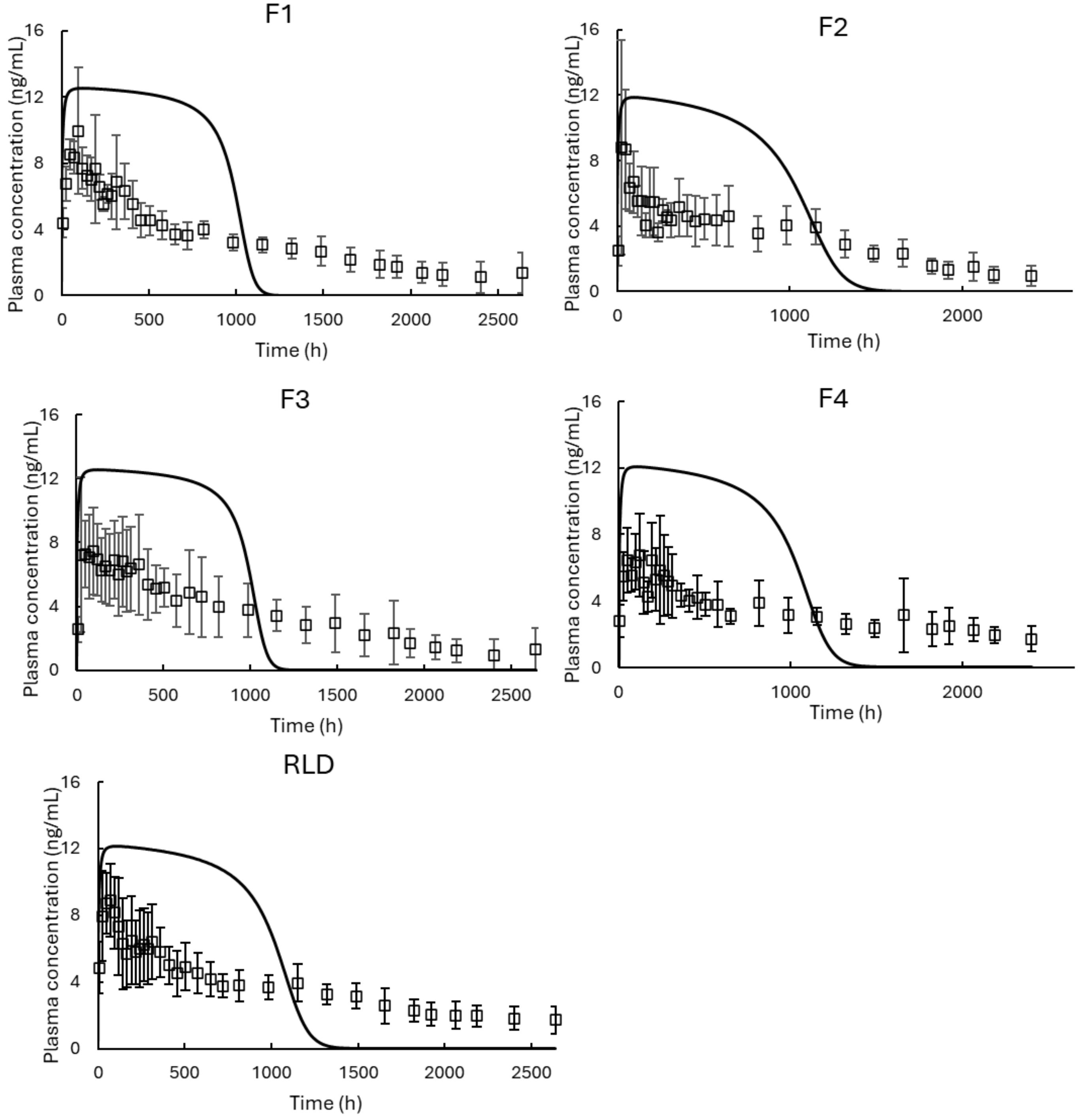

Reference

1. Bao Q, Wang X, Wan B, Zou Y, Wang Y, Burgess DJ. Development of in vitro-in vivo correlations for long-acting injectable suspensions. Int J Pharm. 2023;634:122642.
