# Supplementary material for: Development of Mechanistic In Vitro–In Vivo Extrapolation to Support Bioequivalence Assessment of Long-Acting Injectables
Source: Pharmaceutics. 2024 Apr 19;16(4):552. doi: 10.3390/pharmaceutics16040552 (PMC11054330; doi:10.3390/pharmaceutics16040552)
Supplement: Supplementary file 1 [file pharmaceutics-16-00552-s001.zip › Supplementary material.pdf]

1. **Methods**

1.1 Human predictions - Oral administration

A fit-for-purpose human PBPK model was built using the same approach taken in the preclinical model- distribution in all tissues was described using perfusion limited model, Kps were calculated using Lukacova (default) method, renal clearance was defined as Fup*GFR and the liver clearance estimated by allometric scaling from rabbit intrinsic clearance. The model was validated with oral data obtained from the literature (1). In the study, Stalker and colleagues assessed the bioavailability of MPA from three 500 mg oral formulations, two of which were marketed tablet formulations of MPA (Farlutal® and Provera®) and the third was a granule formulation. The particle sizes differed between the formulations; however, the values were not reported in the publication. Hence, a fitted particle radius of 14 µm, 9.5 µm and 6.3 µm was used in the model for Farlutal®, Provera®, and the generic granule formulation, respectively.

2 Results

2.1 Human predictions - Oral administration

As shown in Figure S2 and Table S1, the model adequately described the human exposure following oral administration. Hence, the model was further applied to SC administration.

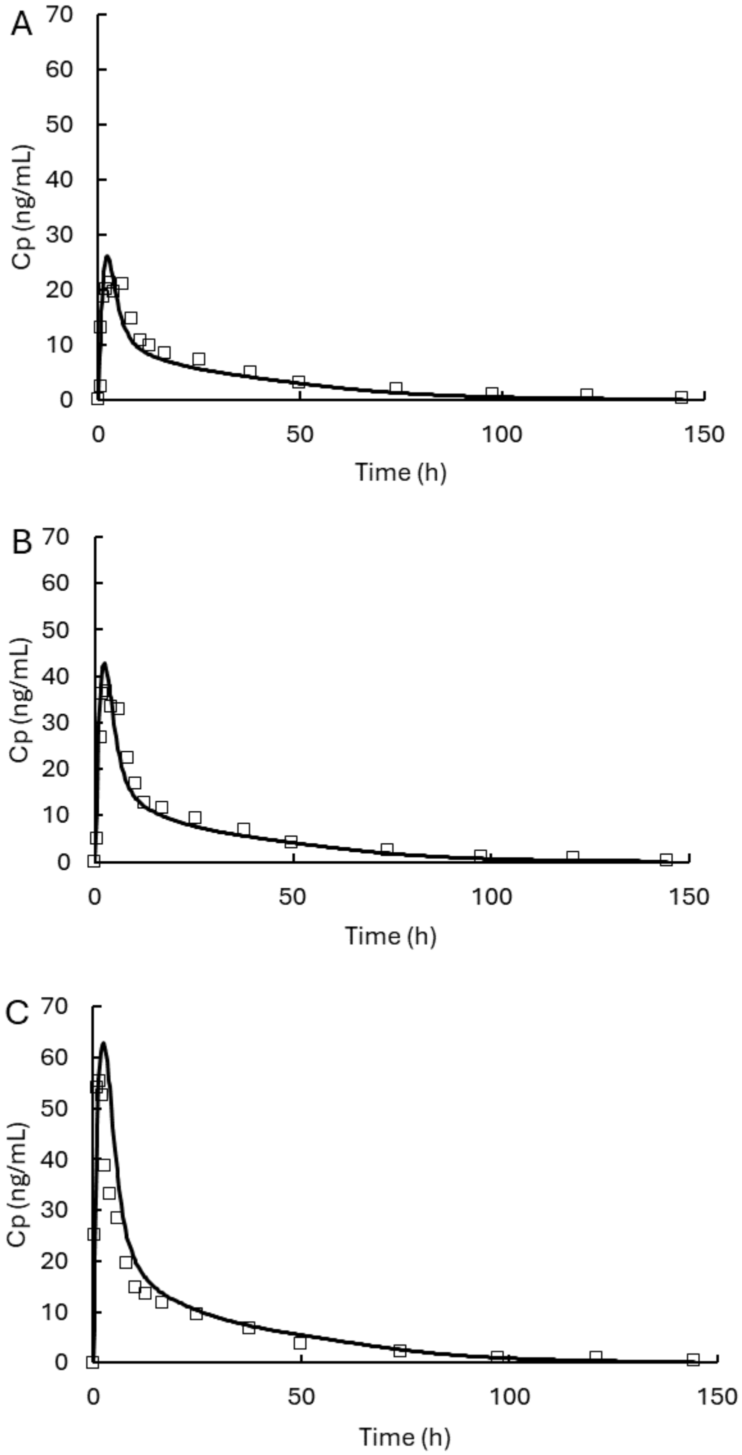

Figure S2. Observed (squares) and simulated (line) plasma MPA concentration after administration of 500 mg Farlutal® tablet (A), 500 mg Provera® tablet (B) and 500 mg Granule formulation (C) in men. Observed data obtained from (1).

Table S1. Comparison of observed and predicted Cmax and AUC after oral MPA administration.

	Farlutal Tablet			Provera Tablet			Provera Granules		
	**Obs.**	**Sim.**	**FE**	**Obs.**	**Sim.**	**FE**	**Obs.**	**Sim.**	**FE**
**Cmax**	21.27	26.128	1.23	36.85	42.664	1.16	55.35	62.857	1.14
**AUC 0-inf**	566.1	450.94	0.80	784	668.73	0.85	812.3	945.09	1.16
**AUC 0-t**	554.9	448.96	0.81	772.8	665.86	0.86	803.8	941.19	1.17

Cmax in ng/mL; AUC in ng-h/mL; FE= Fold-error (Simulated/Observed); Obs: Observed; Sim: Simulated

References

1. Stalker D, Welshman I, Pollock S. Bioavailability of medroxyprogesterone acetate from three oral dosage formulations. Clin Ther. 1992;14(4):544–52.
